# Mitochondrial cytochrome *b* sequence variation and wing geometric morphometrics among *Haematobia exigua* (Diptera: Muscidae) populations in Thailand

**DOI:** 10.1016/j.crpvbd.2026.100446

**Published:** 2026-10-09

**Authors:** Tanasak Changbunjong, Thekhawet Weluwanarak, Sedthapong Laojun, Tanawat Chaiphongpachara

**Affiliations:** a Department of Pre-Clinic and Applied Animal Science, Faculty of Veterinary Science, Mahidol University, Nakhon Pathom, 73170, Thailand; b The Monitoring and Surveillance Center for Zoonotic Diseases in Wildlife and Exotic Animals (MoZWE), Faculty of Veterinary Science, Mahidol University, Nakhon Pathom, 73170, Thailand; c Department of Public Health and Health Promotion, College of Allied Health Sciences, Suan Sunandha Rajabhat University, Samut Songkhram, 75000, Thailand

**Keywords:** Buffalo fly, Genetic diversity, Haplotype network, Nucleotide diversity, Phenotypic variation

## Abstract

The buffalo fly, *Haematobia exigua* (Diptera: Muscidae), is an obligate blood-feeding ectoparasite of livestock that adversely affects animal health and productivity. This study combined mitochondrial cytochrome *b* (*cytb*) sequence analysis and wing geometric morphometrics to investigate genetic differentiation and morphological variation among six *H. exigua* populations sampled from different regions of Thailand. Analysis of 64 *cytb* sequences identified 19 haplotypes, with overall haplotype diversity of 0.725 ± 0.060, nucleotide diversity of 0.00222 ± 0.00032, and an average number of nucleotide differences of 1.317. The median-joining haplotype network exhibited a partially star-like pattern, while the significantly negative neutrality test results for the total dataset indicated an excess of low-frequency variants, a pattern consistent with possible demographic expansion. AMOVA revealed that most genetic variation occurred within populations, with limited variation among populations. The overall *F*_ST_ value was low but significant (*F*_ST_ = 0.037, *P* < 0.05), suggesting weak genetic differentiation among *H. exigua* populations. Wing geometric morphometrics also revealed significant variation among the sampled populations. Pairwise Mahalanobis distances showed significant differences in wing shape among all population pairs (*P* < 0.05). These findings indicate limited mitochondrial differentiation but clear wing-shape variation among the sampled populations. The combined use of genetic and morphometric approaches provides complementary population-level information and establishes a baseline for future studies on the ecology, dispersal, and management of *H. exigua* in Thailand.

## Highlights

• First combined assessment of genetic and wing phenotypic variation in *Haematobia exigua* populations from Thailand.
• Mitochondrial *cytb* analysis revealed high haplotype diversity and significant population differentiation.
• Wing geometric morphometrics revealed significant wing-shape variation among the sampled *H. exigua* populations.

## Graphical abstract

**Figure:**
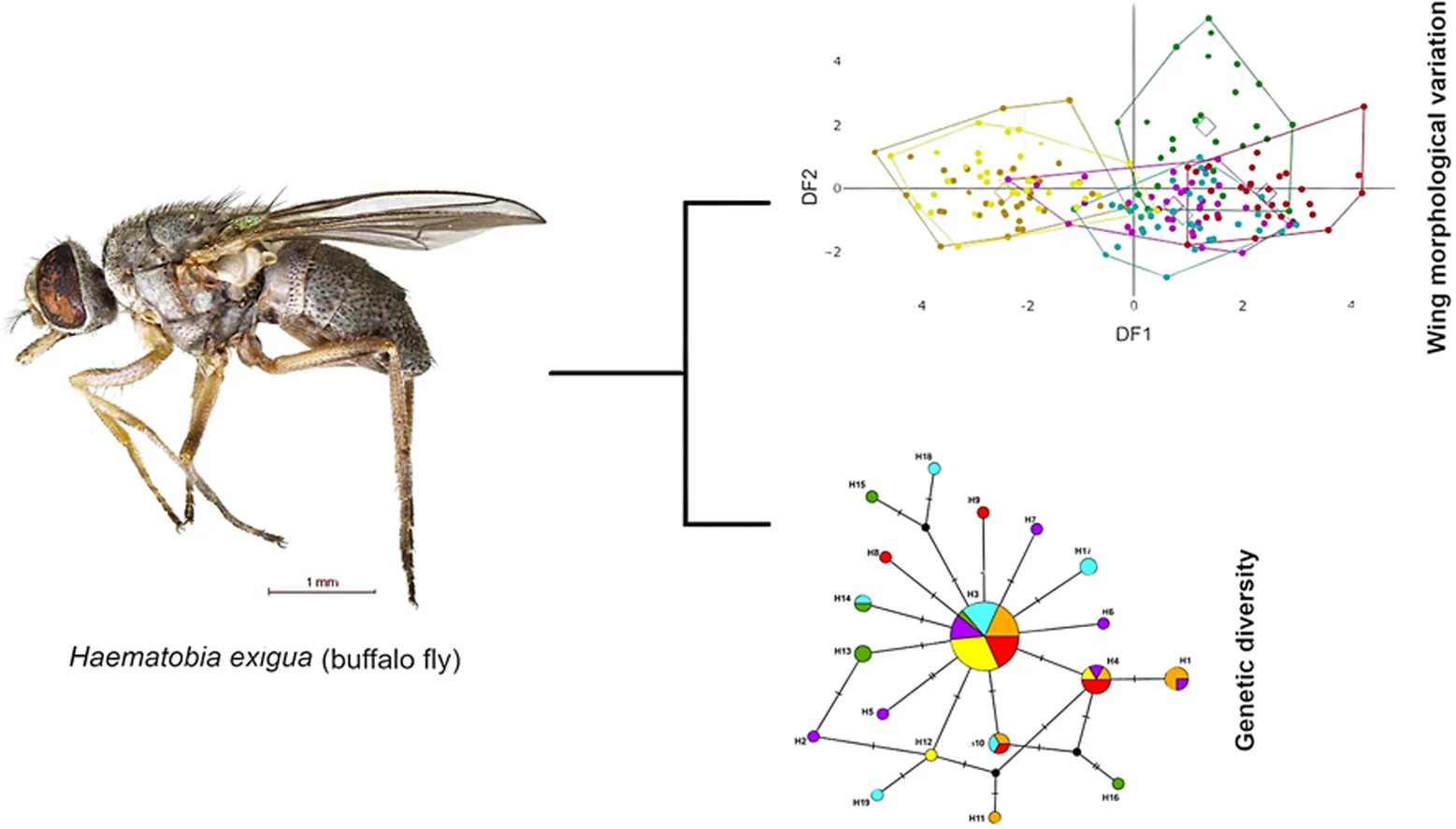

## 1 Introduction

The buffalo fly, *Haematobia exigua* de Meijere, 1903 (Diptera: Muscidae), is an obligate blood-feeding ectoparasite of considerable veterinary importance, particularly in cattle production systems (Jonsson and Mayer, 1999; James et al., 2021). This species is distributed mainly in the Oriental and Australasian regions (Zumpt, 1973; Iwasa and Ishiguro, 2010; Low et al., 2014). Adult males and females require blood meals and spend most of their lives on the host, where they repeatedly feed and rest on the body surface (Macqueen and Doube, 1988). The primary hosts of *H*. *exigua* are water buffalo and cattle, but it is also capable of feeding on other warm-blooded species such as pigs, horses, rabbits, guinea pigs, wildlife, and rarely humans when preferred livestock hosts are not available (Zumpt, 1973; Changbunjong et al., 2012). Severe infestations may cause irritation, hemorrhage, skin lesions, reduced feeding efficiency and stress to the affected animals, resulting in reduced weight gain and lower milk yield (Sutherst and Tozer, 1995; Parashar et al., 2001; James et al., 2021). Beyond their direct effects on animal health and productivity, buffalo flies may also contribute to pathogen transmission. They can transmit *Stephanofilaria* sp., a filarial nematode associated with buffalo fly lesions in cattle (Naseem et al., 2022a). Moreover, *Staphylococcus agnetis* and *Staphylococcus hyicus* have been identified in both buffalo flies and associated skin lesions, suggesting that these bacteria may contribute to lesion pathogenesis (Naseem et al., 2022b).

In Thailand, a Southeast Asian country, *H. exigua* is an important livestock ectoparasite commonly found on dairy and beef cattle (Changbunjong et al., 2012). It has been recorded from several geographical regions, including Kanchanaburi in the west, Nakhon Si Thammarat in the south, Chonburi in the east, and Nakhon Pathom in the central region (Changbunjong et al., 2012; Yeohsakul et al., 2024; Phetcharat et al., 2024). Because this fly species occurs across geographically separated livestock-rearing areas, understanding its population structure is important for clarifying patterns of dispersal, population connectivity, and differentiation. Previous studies of hematophagous insects in Thailand, including mosquitoes (Chaiphongpachara et al., 2024; Laojun et al., 2024; Laojun and Chaiphongpachara, 2025), *Stomoxys* flies (Chaiphongpachara et al., 2022a), and horse flies (Chaiphongpachara et al., 2022b), have shown that geographical variation may be associated with genetic and morphological differentiation. However, information on population structure and wing geometric morphometric variation in *H. exigua* remains limited, both in Thailand and elsewhere.

Assessing genetic differentiation among natural populations can provide useful insights into dispersal patterns, population connectivity, and differentiation among habitats (Landmann et al., 2021; de Araujo Barbosa et al., 2025). Mitochondrial DNA is widely used in population studies because it provides haplotype-level information that can reveal maternal lineage structure and recent demographic patterns (Ballard and Pichaud, 2014; Dong et al., 2021). Its usefulness is related to several characteristics, including maternal inheritance, a relatively high mutation rate, limited recombination, a comparatively stable molecular clock, and suitability for haplotype-based analysis (Avise et al., 1987; Zink and Barrowclough, 2008; Ballard and Pichaud, 2014). Among mitochondrial markers, the cytochrome *b* (*cytb*) gene has been applied to studies of genetic variation and population relationships in biting flies (Low et al., 2014; Changbunjong et al., 2024, 2025). A previous comparative study using multiple molecular markers, including cytochrome *c* oxidase subunit 1 (*cox*1), *cytb*, NADH dehydrogenase subunit 5 (*nad*5), 18S ribosomal RNA (18S rRNA), and 28S ribosomal RNA (28S rRNA), showed that *cytb* was useful for distinguishing *H. exigua* from *H. irritans* and for examining mitochondrial variation within *Haematobia* spp. (Low et al., 2014). Therefore, mitochondrial *cytb* sequences provide useful genetic information for evaluating population structure in *H. exigua*.

In quantitative morphological studies, insect wings are widely used because they have clear geometric structures that can be measured and analyzed systematically (Zelditch et al., 2012; Lorenz et al., 2017). Wings are also functionally important for flight and dispersal, making their variation relevant to population-level studies. In insect vectors, intraspecific variation in wing traits may reflect differences among populations arising from biological processes, ecological conditions, and population responses to different environments (Lorenz and Suesdek, 2020; Chaiphongpachara et al., 2022a). Geometric morphometrics (GM) is a commonly used approach for evaluating such variation because it uses landmark-based coordinate data and multivariate statistical analyses to quantify differences in wing size and shape (Zelditch et al., 2012; Lorenz et al., 2017). Previous studies have applied this technique to stomoxyine flies across different geographical landscapes and detected wing variation associated with local environmental or spatial conditions (Chaiphongpachara et al., 2022a; Chapoopuang et al., 2025).

To characterize genetic differentiation and wing morphological variation among sampled *H. exigua* populations in Thailand, this study combined mitochondrial *cytb* sequence analysis and wing GM. The findings provide baseline information on genetic and phenotypic variation among the sampled populations and may support future ecological and population-level studies of this species.

## 2 Materials and methods

### 2.1 Study sites

Six sampling localities were included in this study, with one farm selected in each province from the six major geographical regions of Thailand (Table 1, Fig. 1): Chiang Mai (Northern; 18°38′09″N, 98°32′07″E), Nakhon Pathom (Central; 14°00′37″N, 99°55′36″E), Nakhon Ratchasima (Northeastern; 14°38′01″N, 101°12′30″E), Kanchanaburi (Western; 14°14′04″N, 99°13′02″E), Chonburi (Eastern; 13°19′08″N, 101°14′09″E), and Narathiwat (Southern; 06°05′09″N, 101°53′06″E). Thus, each geographical region was represented by a single farm/locality. The sampling sites included beef cattle farms, dairy cattle farms, and a buffalo farm. Altitudes among the sampling localities ranged from 11 to 1110 m above sea level.

**Table 1:** Collection details and number of *Haematobia exigua* specimens selected for wing geometric morphometric and *cytb* analyses.

Population	Code	Region	Month/Year	Collection site	Coordinates	Altitude (m)	No. of specimens selected for analysis	
							*cytb*	GM
Chiang Mai	CM	Northern	November 2025	Beef cattle farm	18°38′09″N, 98°32′07″E	1110	M 2, F 4	M 25, F 25
Nakhon Pathom	NP	Central	January 2026	Dairy cattle farm	14°00′37″N, 99°55′36″E	11	M 6, F 6	M 35, F 35
Nakhon Ratchasima	NR	Northeastern	December 2025	Dairy cattle farm	14°38′01″N, 101°12′30″E	232	M 7, F 3	M 35, F 35
Kanchanaburi	KB	Western	January 2026	Beef cattle farm	14°14′04″N, 99°13′02″E	86	M 6, F 6	M 30, F 30
Chonburi	CB	Eastern	April 2026	Buffalo farm	13°19′08″N, 101°14′09″E	39	M 6, F 6	M 35, F 35
Narathiwat	NT	Southern	December 2025	Beef cattle farm	06°05′09″N, 101°53′06″E	28	M 6, F 6	M 35, F 35
Total							M 33, F 31	M 195, F 195

*Note*: The numbers shown represent specimens selected for *cytb* sequencing and GM analysis and do not represent the total number of flies collected at each locality.

*Abbreviations*: M, male; F, female; GM, geometric morphometric analysis; *cytb*, cytochrome *b* gene.

**Fig. 1:**
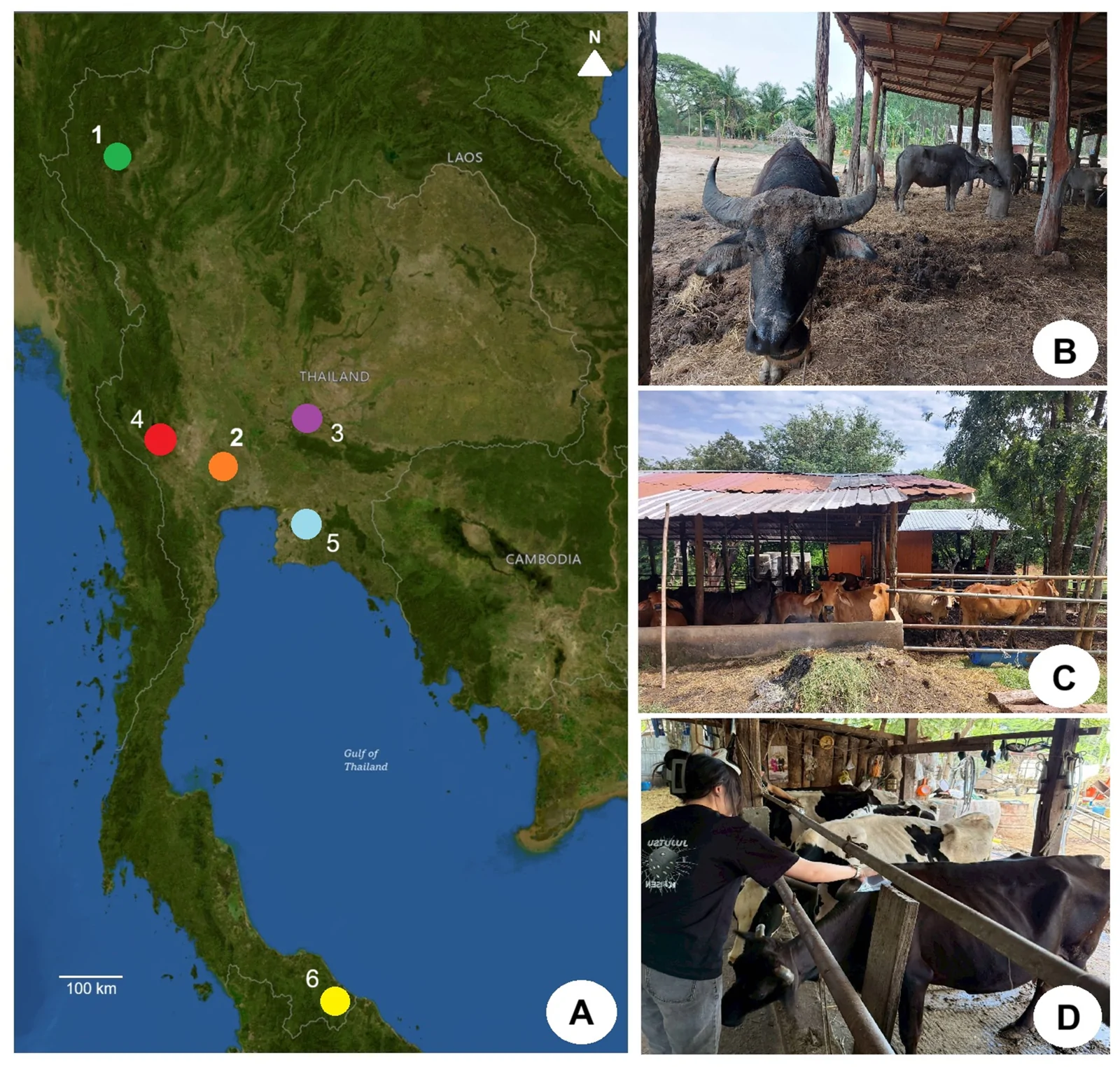
Geographical locations of *Haematobia exigua* collection sites in Thailand. **A** Map showing six collection sites across representative regions of Thailand: 1, Chiang Mai Province, Northern region; 2, Nakhon Pathom Province, Central region; 3, Nakhon Ratchasima Province, Northeastern region; 4, Kanchanaburi Province, Western region; 5, Chonburi Province, Eastern region; and 6, Narathiwat Province, Southern region. **B**-**D** Buffalo (**B**), beef cattle (**C**), and dairy cattle (**D**) representing the host animals from which *H. exigua* specimens were collected. The map was sourced from the USGS National Map Viewer (National Map Viewer, public domain: https://apps.nationalmap.gov/).

The Chiang Mai locality was situated in a mountainous highland area in northern Thailand, whereas Nakhon Pathom was located in a central lowland agricultural area. Nakhon Ratchasima was situated on the northeastern plateau, Kanchanaburi in a western mountainous area with mixed agricultural and forest-edge environments, Chonburi in an eastern lowland area near the Gulf of Thailand, and Narathiwat in a southern tropical coastal lowland area. Detailed information on geographical region, sampling period, farm type, geographical coordinates, altitude, and the number of specimens used for molecular and GM analyses is provided in Table 1.

### 2.2 Sample collection and morphological identification

Adult *Haematobia* spp. flies were collected directly from the body surfaces of cattle and buffalo at the six selected farms between November 2025 and April 2026. At each farm, flies were collected from multiple host animals, and each farm housed more than 10 host animals. Sampling was conducted during the early morning and late afternoon when the animals were confined in pens.

Individual flies resting on the host were collected using 16 × 22 cm ziplock bags, which were carefully placed over the flies before they were enclosed inside. Captured flies were euthanized by freezing at −20 °C using a portable freezer in the field and subsequently transported to the laboratory. Adult male and female specimens were examined using a Nikon AZ100 stereomicroscope (Nikon Corporation, Tokyo, Japan). Morphological identification was based on external characters using the taxonomic key provided by Zumpt (1973). Specimens confirmed as *H. exigua* were placed individually in 1.5-ml microcentrifuge tubes and stored at −20 °C until DNA extraction.

### 2.3 DNA extraction, *cytb* amplification, sequencing, and sequence preparation

Genomic DNA was extracted from the legs of each adult *H*. *exigua* specimen using the FavorPrep™ Tissue Genomic DNA Extraction Mini Kit (Favorgen Biotech, Ping-Tung, Taiwan) according to the manufacturer’s instructions. The extracted DNA was stored at −20 °C until PCR amplification. A partial fragment of the mitochondrial *cytb* gene was amplified using the primer pair: CB-J10933 forward primer (5′-GTT TTA CCT TGA GGA CAA ATA TC-3′) and CB-N11526 reverse primer (5′-TTC AAC TGG TCG AGC TCC AAT TCA-3′) (Simon et al., 1994).

Each PCR was prepared in a total volume of 25 μl. The reaction mixture consisted of 5 μl template genomic DNA (50–100 ng), 1× PCR buffer, 1.5 mM MgCl_2_, 1.5 units of Platinum Taq DNA polymerase (Invitrogen, CA, USA), 0.1 μM of each primer, 0.2 mM dNTPs (Invitrogen, CA, USA) and distilled water to the final volume. PCR cycling consisted of an initial denaturation at 94 °C for 4 min, followed by 35 cycles of denaturation at 94 °C for 40 s, annealing at 58 °C for 40 s, and extension at 72 °C for 1 min, with a final extension step at 72 °C for 10 min. A negative control without DNA template and a positive control containing DNA from an *H. exigua* specimen were included in each PCR run. Amplified fragments were revealed and confirmed by electrophoresis on 1.5% agarose gels stained with Novel Juice DNA stain (GeneDireX, Taiwan), and visualized under an SCG-W1000 Plus gel imaging system (Wuhan Servicebio Technology Co., Ltd., Wuhan, China). A sample was regarded as successfully amplified when a clear single band was observed at the expected fragment size. Finally, positive PCR products were purified and sequenced *via* U2Bio Co., Ltd. (Seoul, South Korea).

Forward and reverse *cytb* chromatograms were inspected, trimmed, and assembled in BioEdit v.7.2.5 (Hall, 1999) to generate a consensus sequence for each specimen. All consensus sequences were trimmed to the same nucleotide region before alignment. The final aligned sequence length was 592 bp for all 64 sequences, and all sequences covered identical nucleotide positions. No sequences were excluded because of incomplete or poor-quality sequence data. Consensus sequences were then compared with available sequences in GenBank using the Basic Local Alignment Search Tool (BLAST) to confirm the identity of *H. exigua*. The *cytb* sequences of *H. exigua* generated in this study were deposited in the GenBank database under the accession numbers PZ631306–PZ631369.

### 2.4 Genetic diversity analysis

Consensus *cytb* sequences obtained from each specimen were aligned using the ClustalW multiple sequence alignment algorithm in MEGA 11 software (Tamura et al., 2021). The population diversity indices: the number of segregating sites (*S*), nucleotide diversity (*π*), number of haplotypes (*h*), haplotype diversity (*Hd*), and the average number of nucleotide differences (*κ*) were calculated using the final alignment with DnaSP v.6 (Rozas et al., 2017). Nucleotide diversity (*π*) is the average number of nucleotide differences per site between two randomly sampled sequences within a population (Tajima, 1983). Haplotypes were defined as distinct nucleotide sequence types, and sequences differing by at least one nucleotide site were considered different haplotypes. Haplotype diversity was used to estimate the probability that two randomly chosen individuals from a population have different haplotypes (Zouré et al., 2020). The relationships of haplotypes among the populations of *H. exigua* from different geographic regions of Thailand were illustrated by the median-joining algorithm in PopART v.1.7 (Leigh and Bryant, 2015).

### 2.5 Demographic history analysis

Neutrality tests were performed using DnaSP to examine departures from neutral expectations in each population and in the total dataset. Tajima’s D, Fu’s Fs, Fu & Li’s D, and Fu & Li’s F were calculated to examine departures from neutral expectations. Tajima's D compares estimates of genetic variation based on segregating sites and pairwise nucleotide differences, while Fu’s Fs evaluates deviations from neutrality based on haplotype distribution. Fu & Li’s D and F were included as additional neutrality tests based on biallelic positions, which refer to variable nucleotide sites with two observed nucleotide variants in the sequence alignment. Significant negative values were interpreted as possible signals of demographic expansion or an excess of rare variants. Statistical significance was assessed at *P* < 0.05.

### 2.6 Population structure analysis

The genetic structure among *H. exigua* populations was assessed using analysis of molecular variance (AMOVA) in Arlequin v.3.5 (Excoffier and Lischer, 2010). AMOVA was performed to partition genetic variation into among-population and within-population components among the six sampled populations. Molecular distances were calculated using pairwise differences based on *cytb* sequences, and the statistical significance of variance components was tested using 10,000 permutations. Pairwise genetic differentiation among populations was calculated using the fixation index (*F*_ST_). Significance of pairwise *F*_ST_ values was evaluated with 10,000 permutations.

### 2.7 Wing geometric morphometric analysis

Adult *H*. *exigua* specimens representing the six geographical regions of Thailand were selected at random for wing GM analysis. A subset of 390 adult *H. exigua* specimens was selected for wing GM analysis, including all 64 specimens used for molecular analysis. The left wing of each specimen was carefully removed and mounted on a microscope slide using Hoyer’s medium. Specimens with damaged wings were excluded from the analysis. Mounted wings were photographed using an imaging system connected to a Nikon AZ100 stereomicroscope. Images were calibrated using a 1-mm scale. Each image was labelled with a unique code containing information on the sampling site, locality, sex, and specimen number. These images were then used for the landmark-based GM analysis in XY Online Morphometrics (Dujardin and Dujardin, 2019).

For landmark digitization, each wing was analyzed using ten homologous landmarks (Fig. 2). The landmarks were at the wing vein intersections and at the points where the veins join the wing margin. The landmark configuration was based on previous studies on stomoxyine flies where wing venation landmarks were efficiently used to evaluate differences in wing size and shape and differences at the population level (Changbunjong et al., 2016, 2023). Before the size and shape analyses, digitizing error was evaluated to ensure the precision of landmark digitization based on a repeatability test. Ten wing images were randomly selected and digitized twice by the same operator. Repeatability was then assessed using Procrustes analysis of variance, and the repeatability index (R) was calculated following Arnqvist and Mårtensson (1998).

**Fig. 2:**
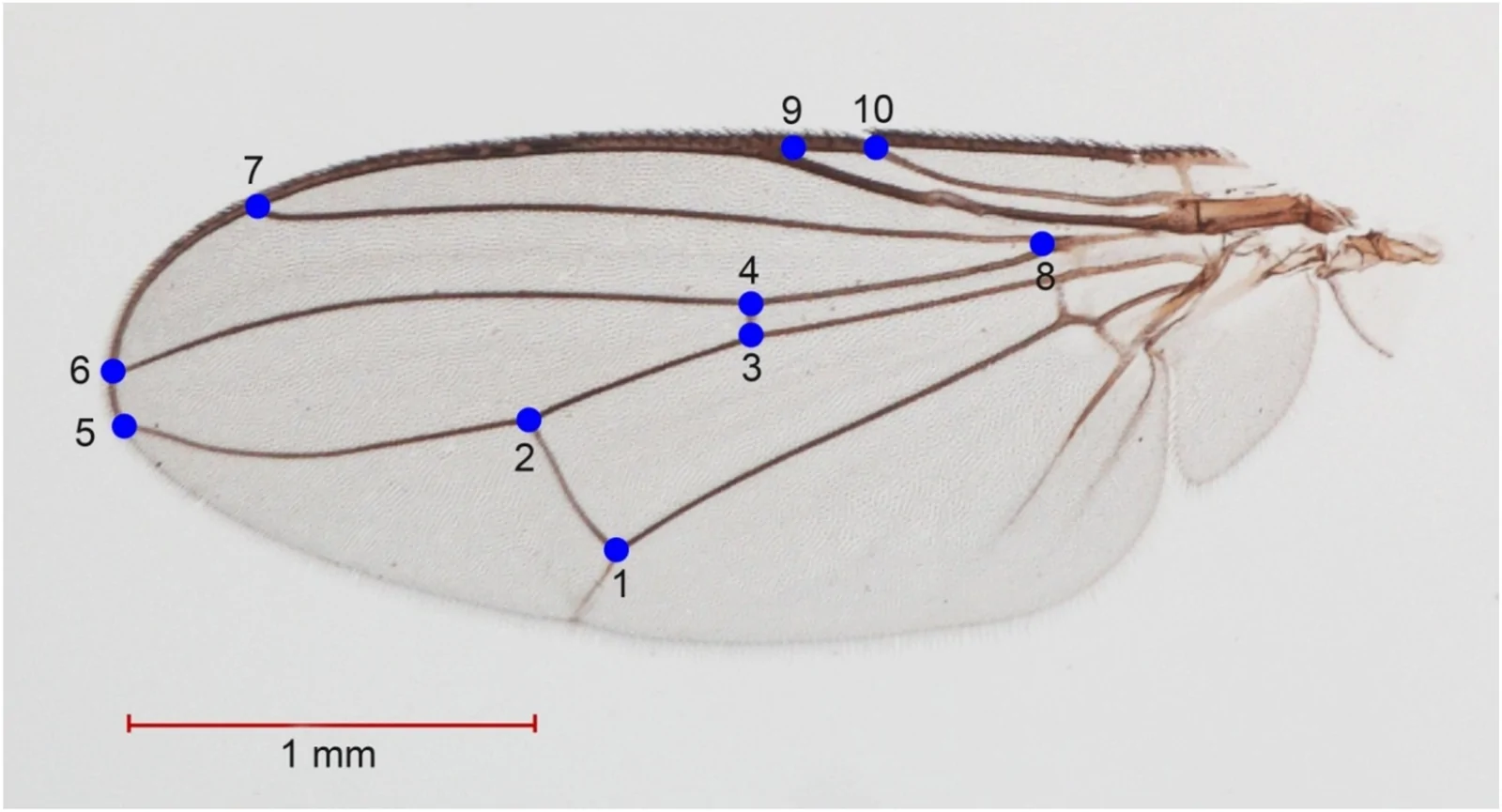
Ten anatomical landmarks on the left wing of *Haematobia exigua* used for wing geometric morphometric analysis. *Scale-bar*: 1 mm.

Wing size of *H. exigua* was estimated using centroid size (CS), which was calculated from the square root of the sum of squared distances of all landmarks from the centroid of the landmark configuration. Differences in centroid size among the 12 population-by-sex groups were evaluated using one-way ANOVA. Pairwise comparisons were then performed using non-parametric permutation tests with 1000 replicates. After that, allometry was evaluated by regressing the first principal component of wing shape (PC1) against wing CS. The relationship was quantified using the Pearson’s correlation coefficient (*r*) and coefficient of determination (*R*^2^). As the association was weak, the original shape data were retained for subsequent analyses without allometric correction.

Wing shape variables were obtained from Procrustes residuals (orthogonal projections) generated by generalized Procrustes analysis (GPA). Principal components analysis (PCA) was subsequently performed to summarize wing shape variation, and the first 16 principal components (PCs) were retained as shape variables for subsequent analyses. Discriminant analysis (DA) was performed to evaluate wing shape differentiation among geographical populations. The relationships among populations were visualized using factor maps based on the first two discriminant factors (DFs). Shape differences among geographical populations were quantified using Mahalanobis distances, and their significance was assessed using non-parametric permutation tests with 1000 replicates after Bonferroni correction for multiple pairwise comparisons. Statistical significance of all GM analyses was set at *P* < 0.05.

## 3 Results

### 3.1 Genetic diversity

Sequence comparison against GenBank confirmed the identity of the specimens as *H. exigua*. All 64 mitochondrial *cytb* sequences showed 99–100% identity with reference sequences identified as *H. exigua* in GenBank. The closest BLAST matches and corresponding reference accession numbers are provided in Supplementary Table S1. A total of 64 mitochondrial *cytb* sequences were obtained from six populations in Thailand (Table 2). The final dataset contained 19 haplotypes and 20 segregating sites. The *cytb* sequences were AT-rich in nucleotide composition (A = 32.30%; T = 43.39%; C = 13.03%; and G = 11.28%).

**Table 2:** Genetic diversity indices of *Haematobia exigua* populations in Thailand based on *cytb* sequences.

Population	Region	*n*	GenBank ID	*S*	*π* ± SD	*h*	*Hd* ± SD	*κ*
Chiang Mai	Northern	6	PZ631352-PZ631357	8	0.00484 ± 0.00139	5	0.933 ± 0.122	2.867
Nakhon Pathom	Central	12	PZ631328-PZ631339	5	0.00243 ± 0.00055	5	0.727 ± 0.113	1.440
Nakhon Ratchasima	Northeastern	10	PZ631306-PZ631315	8	0.00297 ± 0.00071	7	0.867 ± 0.107	1.756
Kanchanaburi	Western	12	PZ631316-PZ631327	4	0.00154 ± 0.00036	5	0.727 ± 0.113	0.909
Chonburi	Eastern	12	PZ631358-PZ631369	7	0.00220 ± 0.00061	6	0.758 ± 0.122	1.303
Narathiwat	Southern	12	PZ631340-PZ631351	2	0.00056 ± 0.00031	3	0.318 ± 0.164	0.333
Total data estimates		64	PZ631306-PZ631369	20	0.00222 ± 0.00032	19	0.725 ± 0.060	1.317

*Abbreviations*: *n*, number of sequences; *S*, number of segregating sites; *π*, nucleotide diversity; SD, standard deviation; *h*, number of haplotypes; *Hd*, haplotype diversity; *κ*, average number of nucleotide differences.

Genetic diversity indices are presented in Table 2. For the total dataset, the average number of nucleotide differences was 1.317, haplotype diversity was 0.725 ± 0.060, and nucleotide diversity was 0.00222 ± 0.00032. Among the six populations, the Chiang Mai population had the highest values for nucleotide diversity (*π* = 0.00484 ± 0.00139), haplotype diversity (*Hd* = 0.933 ± 0.122), and average number of nucleotide differences (*κ* = 2.867), whereas the Narathiwat population had the lowest values for these indices (*π* = 0.00056 ± 0.00031; *Hd* = 0.318 ± 0.164; and *κ* = 0.333). The highest number of haplotypes was found in the population from Nakhon Ratchasima, with seven haplotypes from ten sequences.

### 3.2 Haplotype network based on the mitochondrial *cytb* gene

The median-joining network constructed from 64 *cytb* sequences identified 19 haplotypes (H1-H19; Fig. 3). H3 was the most frequent haplotype (*n* = 33) and was shared among all six geographical regions: Central (*n* = 6), Eastern (*n* = 6), Northern (*n* = 1), Northeastern (*n* = 4), Southern (*n* = 10), and Western (*n* = 6). Several haplotypes were connected to H3 or closely related haplotypes by only a few mutational steps, resulting in a star-like network pattern. Both region-specific and shared haplotypes were observed. Region-specific haplotypes included H2 and H5-H7 in the Northeastern population, H8-H9 in the Western population, H11 in the Central population, H12 in the Southern population, H13 and H15-H16 in the Northern population, and H17-H19 in the Eastern population. Haplotypes H1, H4, H10, and H14 were shared between two or more regions.

**Fig. 3:**
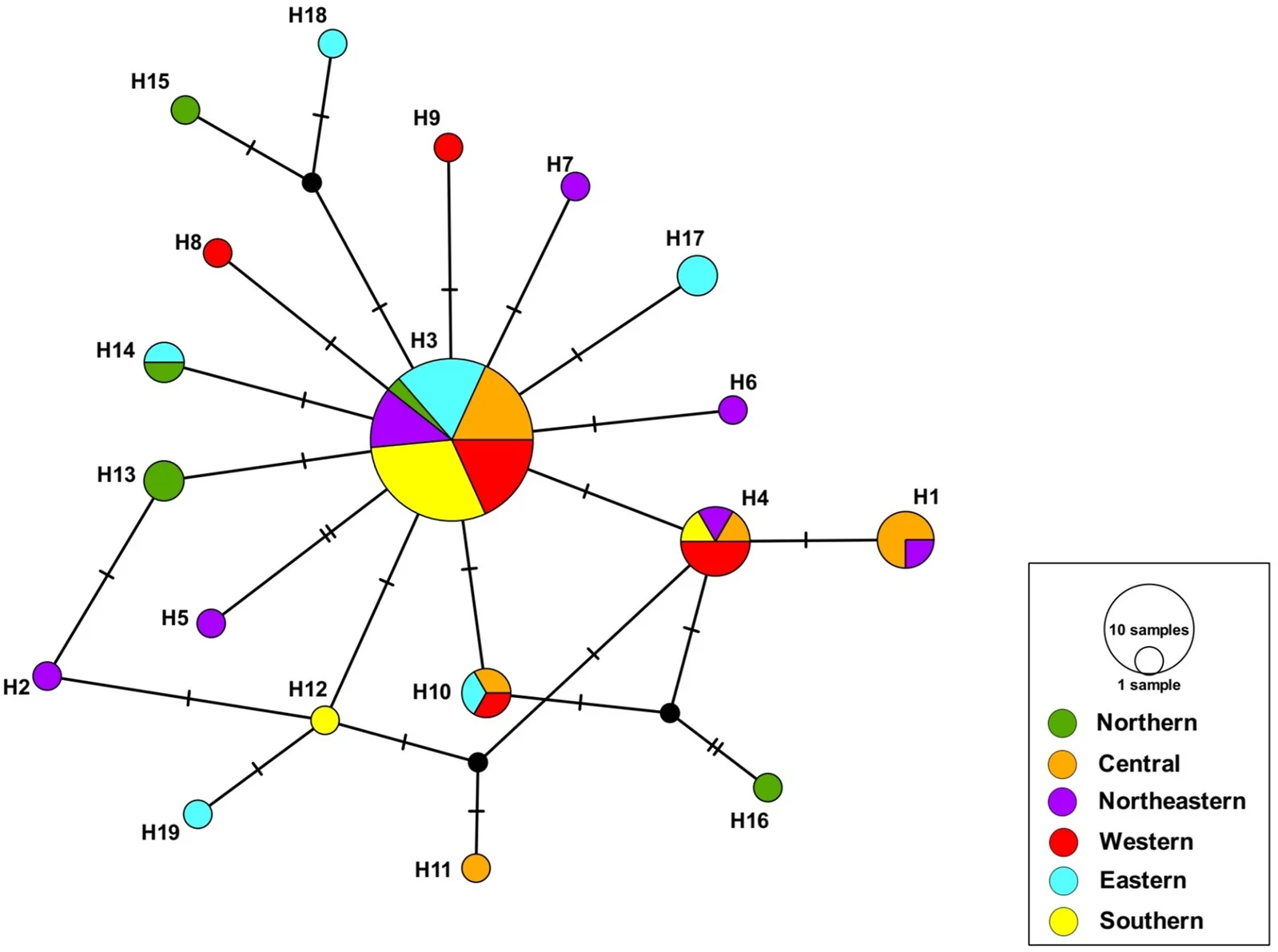
Median-joining haplotype network for *Haematobia exigua* populations in Thailand based on *cytb* sequences. Haplotype circles are proportional in size to the number of individuals sharing each haplotype. Different colors indicate the geographical distribution of haplotypes among the six populations; black circles represent unsampled haplotypes; hatch marks represent the number of mutational steps.

### 3.3 Demographic history inferred from neutrality tests

All four neutrality tests, including Tajima’s D, Fu’s Fs, Fu & Li’s D, and Fu & Li’s F, showed negative values across all six populations and the overall dataset (Table 3). For the total dataset, all neutrality statistics were significantly negative, indicating an excess of rare variants. At the population level, most neutrality test values were not statistically significant (*P* > 0.05). Among individual populations, only Fu’s Fs in the Nakhon Ratchasima population showed a significant negative value (Fu’s Fs = −3.711, *P* < 0.05).

**Table 3:** Neutrality tests of *Haematobia exigua* populations in Thailand based on *cytb* sequences.

Population	Region	Neutrality tests			
		Tajima’s D	Fu’s Fs	Fu & Li’s D	Fu & Li’s F
Chiang Mai	Northern	−1.072	−1.372	−1.063	−1.145
Nakhon Pathom	Central	−0.482	−1.002	−0.833	−0.842
Nakhon Ratchasima	Northeastern	−1.636	−3.711*	−1.733	−1.92
Kanchanaburi	Western	−1.103	−2.144	−1.286	−1.402
Chonburi	Eastern	−1.713	−2.524	−1.855	−2.062
Narathiwat	Southern	−1.451	−1.325	−1.72	−1.865
Total data estimates		−2.116*	−16.708*	−3.064*	−3.242*

*Note*: An asterisk (*) indicates statistical significance at *P* < 0.05.

### 3.4 Population genetic structure

AMOVA showed that most genetic variation in *H. exigua* occurred within populations, whereas only a small proportion was attributed to differences among populations. The overall *F*_ST_ value was low but statistically significant (*F*_ST_ = 0.037, *P* < 0.05; Table 4), indicating weak but detectable genetic structuring. Pairwise *F*_ST_ comparisons were generally low (Table 5). Significant genetic differentiation was observed only between the Central and Eastern populations (*F*_ST_ = 0.119, *P* < 0.05) and between the Northern and Southern populations (*F*_ST_ = 0.118, *P* < 0.05). Several population pairs exhibited *F*_ST_ values close to zero or negative, suggesting little or no detectable genetic differentiation among these populations.

**Table 4:** Analysis of molecular variance (AMOVA) among *Haematobia exigua* populations from six sampling sites in Thailand based on *cytb* sequences.

Source of variation	Degrees of freedom	Sum of squares	Variance components	Percentage of variation	*F*_ST_	*P*-value
Among populations	5	4.501	0.025	3.75	0.037	0.032*
Within populations	58	36.983	0.638	96.25		
Total	63	41.484	0.663	100		

*Note*: An asterisk (*) indicates statistical significance at *P* < 0.05.

**Table 5:** Pairwise genetic differentiation (*F*_ST_) among six *Haematobia exigua* populations in Thailand based on *cytb* sequences.

	Population	1	2	3	4	5
1	Chiang Mai (Northern)	–				
2	Nakhon Pathom (Central)	0.078	–			
3	Nakhon Ratchasima (Northeastern)	−0.002	−0.007	–		
4	Kanchanaburi (Western)	0.055	0.017	−0.020	–	
5	Chonburi (Eastern)	0.036	0.119*	0.016	0.041	–
6	Narathiwat (Southern)	0.118*	0.114	−0.016	0.006	0.002

*Note*: Higher *F*_ST_ values indicate greater genetic differentiation between population pairs. An asterisk (*) indicates statistically significant genetic differentiation at *P* < 0.05.

### 3.5 Wing size variation

A total of 390 wings, comprising 195 males and 195 females, were subjected to landmark digitization for GM analyses (Table 1). The repeatability test showed a digitizing error of 1% for wing shape and an overall repeatability score of 99%, indicating high precision in landmark placement. Therefore, the coordinate data were retained without correction and used for subsequent analyses of wing size and shape.

Wing CS varied among the 12 population-sex groups (Table 6, Fig. 4). Among males, specimens from Chiang Mai had the largest mean centroid size (2.63 mm), followed by specimens from Nakhon Ratchasima (2.54 mm), whereas specimens from Nakhon Pathom had the smallest mean value (2.17 mm). Among females, the largest mean CS was also recorded in specimens from Chiang Mai (2.87 mm), while the smallest was observed in specimens from Narathiwat (2.33 mm). Chiang Mai females had significantly larger wings than those of all other groups (*P* < 0.05). Among males, the Chiang Mai population differed significantly from most other populations (*P* < 0.05), but not from Nakhon Ratchasima (*P* > 0.05).

**Table 6:** Descriptive statistics of wing centroid size in male and female *Haematobia exigua* populations from six regions of Thailand.

Population	Region	*n*	Mean (mm)	Range (Min–Max)	Variance	SD
**Male**						
Chiang Mai	Northern	25	2.63^A^	2.25–2.92	0.03	0.16
Nakhon Pathom	Central	35	2.17^E^	1.96–2.42	0.01	0.11
Nakhon Ratchasima	Northeastern	35	2.54^AC^	2.13–2.84	0.03	0.18
Kanchanaburi	Western	30	2.51^CD^	2.26–2.76	0.02	0.16
Chonburi	Eastern	35	2.43^BD^	2.15–2.67	0.02	0.13
Narathiwat	Southern	35	2.19^E^	1.96–2.46	0.02	0.13
**Female**						
Chiang Mai	Northern	25	2.87^F^	2.48–3.20	0.04	0.19
Nakhon Pathom	Central	35	2.36^B^	2.19–2.64	0.01	0.11
Nakhon Ratchasima	Northeastern	35	2.56^AC^	2.29–2.85	0.02	0.14
Kanchanaburi	Western	30	2.57^AC^	2.17–2.98	0.02	0.15
Chonburi	Eastern	35	2.42^BD^	2.10–2.75	0.02	0.14
Narathiwat	Southern	35	2.33^B^	1.94–2.63	0.02	0.13

*Note*: Different superscript letters in Column 4 indicate significant differences among all 12 population-sex groups, including both males and females, at *P* < 0.05.

*Abbreviations*: *n*, number of specimens; SD, standard deviation.

**Fig. 4:**
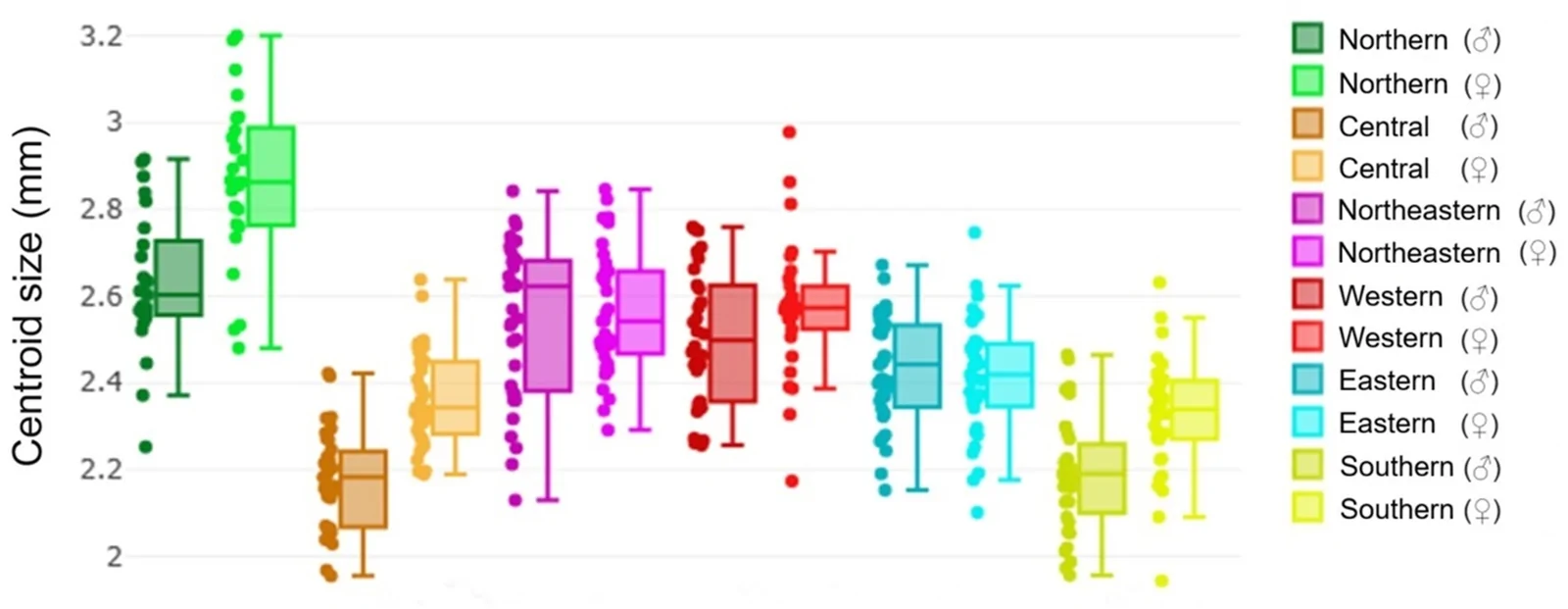
Boxplots of wing centroid size variation in male and female *Haematobia exigua* from six regions of Thailand. Points represent individual specimens; the central line indicates the median, and the lower and upper boundaries of each box represent the 25th and 75th percentiles, respectively.

Regression of the PC1 on CS showed a very weak relationship (*R*^2^ = 0.008, *r* = −0.09; Fig. 5). The nearly horizontal regression line indicated that centroid size explained only a small proportion of the variation in PC1 scores. Therefore, no allometric correction was applied, and the original shape data were retained for subsequent analyses.

**Fig. 5:**
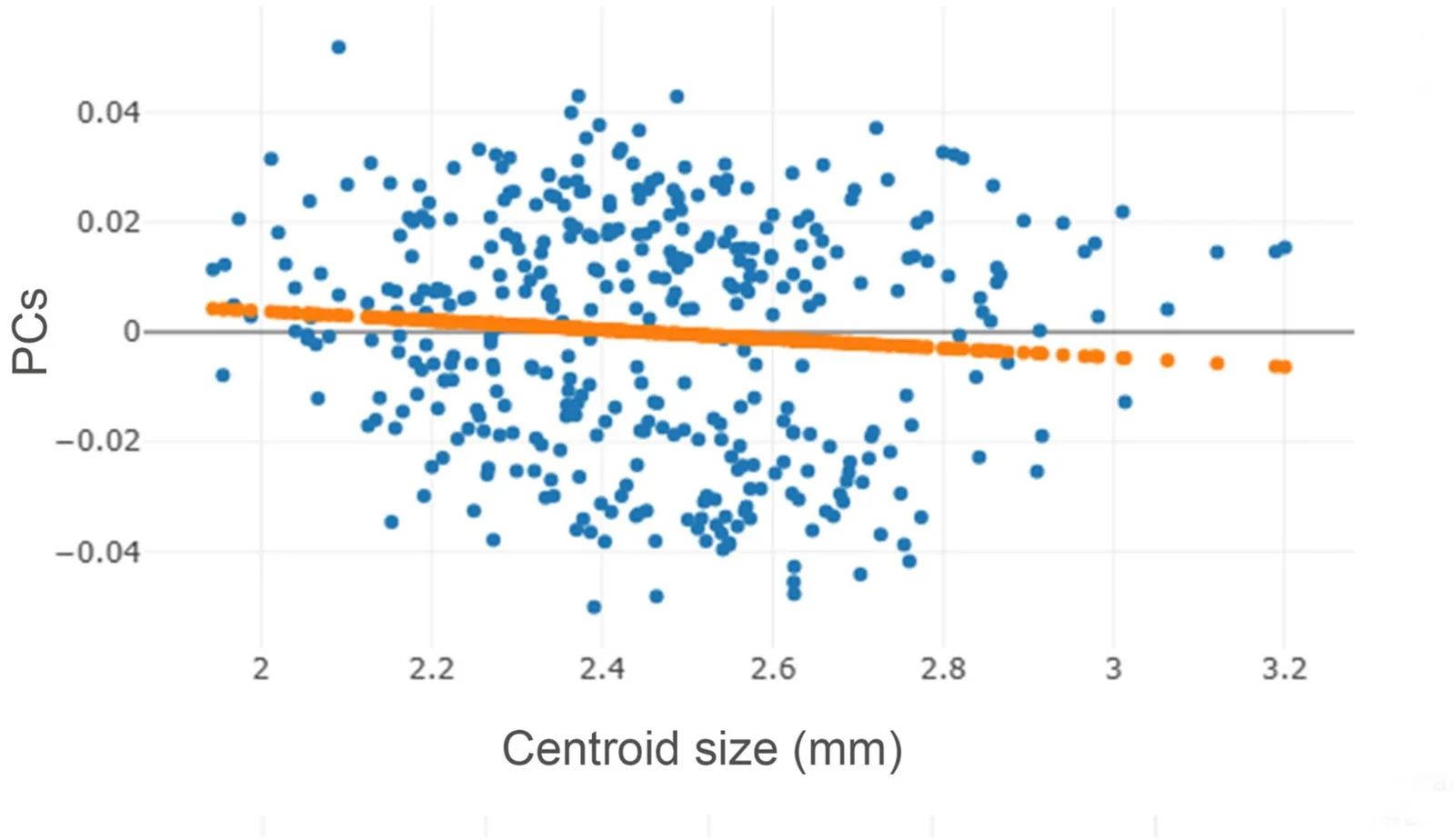
Allometric relationship between wing shape and centroid size in *Haematobia exigua*, based on the regression of the first principal component of shape (PC1) on centroid size. Blue points represent individual specimens, and the orange line represents the fitted regression.

### 3.6 Wing shape variation

Discriminant analysis showed considerable overlap among the six populations in both sexes, with no clear separation among geographical groups (Fig. 6). In males, DF1 and DF2 accounted for 68.2% and 14.2% of the discriminant variation, respectively, while the corresponding values in females were 58.3% and 17.8%. Despite this overlap, differences in group centroid positions indicated subtle variation in wing shape among populations. Pairwise Mahalanobis distances revealed significant differences in wing shape among all population pairs (*P* < 0.05; Table 7). In both sexes, the greatest distance was observed between populations from Nakhon Pathom and Kanchanaburi, with values of 5.01 in males and 3.71 in females. The smallest distance occurred between populations from Nakhon Pathom and Narathiwat, with values of 1.74 and 1.95, respectively.

**Fig. 6:**
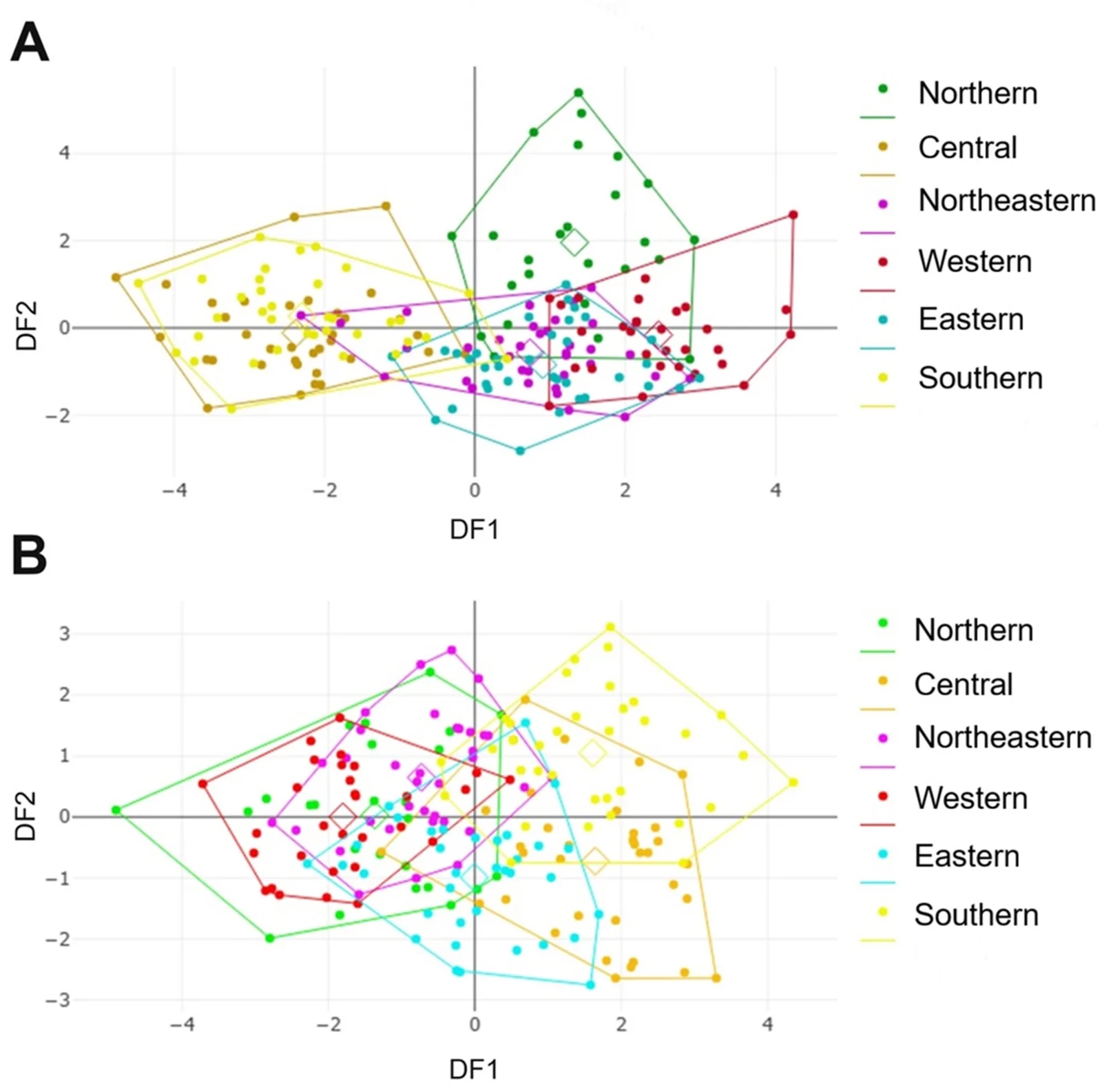
Factor maps of wing shape variation among male and female representatives of *Haematobia exigua* populations from six regions of Thailand. **A** Factor map for males based on 16 principal components, with DF1 and DF2 accounting for 68.2% and 14.2% of the discriminant variation, respectively. **B** Factor map for females based on 16 principal components, with DF1 and DF2 accounting for 58.3% and 17.8% of the discriminant variation, respectively. Points represent individual specimens, polygons indicate the distribution of each regional population, and diamonds represent group centroids.

**Table 7:** Pairwise Mahalanobis distances in wing shape among six *Haematobia exigua* populations in Thailand, with males and females analyzed separately.

	Population	1	2	3	4	5
	**Male**					
1	Chiang Mai (Northern)	–				
2	Nakhon Pathom (Central)	4.31	–			
3	Nakhon Ratchasima (Northeastern)	2.83	3.34	–		
4	Kanchanaburi (Western)	2.83	5.01	2.26	–	
5	Chonburi (Eastern)	2.96	3.67	1.97	2.60	–
6	Narathiwat (Southern)	4.23	1.74	3.48	4.86	3.74
	**Female**					
1	Chiang Mai (Northern)	–				
2	Nakhon Pathom (Central)	3.39	–			
3	Nakhon Ratchasima (Northeastern)	2.04	2.92	–		
4	Kanchanaburi (Western)	2.35	3.71	2.08	–	
5	Chonburi (Eastern)	2.51	2.04	2.00	2.45	–
6	Narathiwat (Southern)	2.52	1.95	2.65	3.69	2.69

*Note*: Larger Mahalanobis distance values indicate greater differences in wing shape between population pairs. All pairwise comparisons were significantly different (*P* < 0.05).

## 4 Discussion

To our knowledge, this study provides the first combined assessment of genetic and phenotypic variation among *H. exigua* populations in Thailand. Analyses of the mitochondrial *cytb* gene and wing GM revealed variation among populations from different regions of the country. Molecular identification confirmed that all 64 specimens included in the *cytb* analysis were *H. exigua*. This finding is consistent with previous studies showing that the *cytb* gene is an effective molecular marker for species identification within the genus *Haematobia* (Low et al., 2014, 2017). The sequences were strongly AT-rich, with an overall AT content of 75.69% compared with a GC content of 24.31%, which is consistent with the nucleotide composition commonly reported for insect mitochondrial DNA (Saito et al., 2005).

Although 19 haplotypes were identified, overall haplotype diversity was relatively high, whereas nucleotide diversity and the average number of nucleotide differences were low, with the latter reaching only 1.317. This pattern indicates that the *cytb* dataset contained multiple haplotypes that were nevertheless separated by only limited sequence divergence. Patterns of genetic variability within populations can provide insight into population dynamics and evolutionary history. Grant and Bowen (1998) proposed that the relationship between haplotype diversity (*Hd*) and nucleotide diversity (*π*) can provide insight into population history. Low *Hd* and low *π* may indicate a recent population bottleneck or founder effect. High *Hd* with low *π* is often associated with rapid population expansion after a past reduction in population size, where newly formed haplotypes differ by only a few mutations. Low *Hd* with high *π* may result from the admixture of previously isolated populations or a decline in a formerly large population. High values of both indices generally characterize a large and historically stable population. Based on this framework, the diversity pattern observed in *H. exigua* is consistent with a possible history of demographic expansion in Thailand.

The median-joining haplotype network further illustrated the genetic relationships among populations. Haplotype H3 was the most frequent haplotype and was shared across all sampled regions, indicating that populations from different parts of Thailand possess a common mitochondrial haplotype. Several low-frequency haplotypes were connected to H3 by only a few mutational steps, producing a star-like network. A similar pattern has been reported for the stable flies *Stomoxys bengalensis* and *Stomoxys sitiens* from central Thailand based on mitochondrial *cox*1 sequences (Mongkolphan et al., 2023). In population genetic studies, a star-like network may be consistent with demographic expansion, although similar patterns can also arise from other demographic or evolutionary processes (Slatkin and Hudson, 1991; Posada and Crandall, 2001). The neutrality tests showed a similar pattern. Although all four statistics were significantly negative for the overall dataset, most population-level values were not significant, with only Fu’s Fs for the Nakhon Ratchasima population showing a significant negative value. Taken together, the star-like haplotype network and neutrality test results are consistent with possible demographic expansion of *H. exigua* in Thailand, but do not provide specific evidence for it. A similar pattern was reported in a broader study of *H. exigua* in Southeast Asia (Low et al., 2017). Populations from the Southeast Asian mainland and Borneo showed evidence of historical population expansion, estimated to have occurred approximately 98,000 years ago during the late Pleistocene. This expansion may have been associated with regional climatic changes that affected habitat suitability and connectivity, as well as the distribution of livestock hosts (Low et al., 2017). During unfavorable periods, populations may have persisted in limited areas and later increased and spread more widely when environmental conditions improved. The effects of past climatic events were not limited to *Haematobia* spp. flies. Similar signals of possible recent demographic expansion have also been reported in Southeast Asian mosquito vectors, including *Armigeres subalbatus* (Laojun et al., 2024) and *Aedes albopictus* (Laojun and Chaiphongpachara, 2025).

AMOVA showed that most genetic variation occurred within populations. The overall *F*_ST_ was low but statistically significant, indicating weak but detectable population structure among the sampled populations. Pairwise *F*_ST_ values were generally low, and several negative values indicated little or no detectable differentiation between most population pairs. This pattern, together with the sharing of haplotypes among several populations, is consistent with limited mitochondrial differentiation among the sampled populations. Significant differentiation was observed only between the Central and Eastern populations and between the Northern and Southern populations, indicating that detectable genetic separation was restricted to certain population pairs. Livestock movement could potentially contribute to gene flow among populations by facilitating the dispersal of *H. exigua*, but this remains a hypothesis because the present mitochondrial dataset does not directly assess host movement or dispersal. Similar regional genetic differences have been reported in several mosquito species (Laojun et al., 2024; Laojun and Chaiphongpachara, 2025), leaf beetle (Kaewmungkoon et al., 2025), and melon fly (Kunprom and Pramual, 2018) in Thailand. Geographical and environmental factors may contribute to population differentiation, although their specific effects could not be evaluated in the present study. In addition, the number of *cytb* sequences differed among populations, with 6–12 sequences available from each locality. This variation in sample size may influence estimates of haplotype diversity and *F*_ST_, as well as the sensitivity of neutrality tests at the population level. These results should therefore be considered in relation to the number of sequences available for each population.

Geometric morphometric analyses of wing size and shape also revealed variation among *H. exigua* populations. Differences in wing size among populations and between sexes may reflect the combined influence of biological and environmental factors (Jeratthitikul et al., 2014; Changbunjong et al., 2020). Females tended to have larger wings than males in several populations, which may be related to differences in reproductive biology and the energetic demands of egg production (Changbunjong et al., 2020). Wing size in insects can also be influenced by environmental conditions during immature development, including temperature, humidity, food availability, larval density, and breeding-site quality (Baleba et al., 2019; Chaiphongpachara et al., 2022a). A recent study on *S. calcitrans* reported that temperature affected wing size, with lower temperatures associated with slower larval development and larger adult wings, whereas higher temperatures were associated with faster development and smaller wings (Chapoopuang et al., 2025). In the present study, however, sampling was conducted in different months at different localities, and the environmental and developmental variables discussed above were not measured directly. Therefore, their specific effects on wing size could not be evaluated or separated from geographical and seasonal variation. The observed differences in wing size should thus be regarded as variation among the sampled populations, while geographical, environmental, and seasonal factors remain possible explanations rather than demonstrated causes.

Wing shape also showed clear variation among populations. Although the factor maps based on DA showed substantial overlap, the significant pairwise Mahalanobis distances indicated that the wing shapes differed among populations. This pattern suggests that the populations were not separated into distinct phenotypic groups, but still showed subtle and consistent differences in wing shape. A similar pattern was reported for *S. calcitrans* populations from different regions of Thailand, where significant shape differences were detected among all populations examined (Chaiphongpachara et al., 2022a). Similar wing-shape variation has been associated with geographical, environmental, and developmental conditions in previous studies (Chaiphongpachara et al., 2022a; Laojun et al., 2024; Laojun and Chaiphongpachara, 2025). However, these factors were not measured directly in the present study, and their specific contribution to the observed wing-shape differences could not be evaluated. The observed pattern should therefore be regarded as phenotypic variation among the sampled populations rather than as evidence of a specific environmental or developmental mechanism.

The effects of host type on the observed genetic and wing-shape variation could not be distinguished from those of sampling locality because each geographical region was represented by a single farm. The mitochondrial *cytb* gene reflects maternally inherited variation and aspects of population history (Yamamoto et al., 2013), whereas wing shape is a quantitative trait influenced by both genetic background and developmental conditions (Alves et al., 2016; Carreira et al., 2011). The two approaches therefore provide complementary information on genetic and phenotypic variation among *H. exigua* populations. Similar influences of local environmental and developmental conditions on wing morphology have also been reported in other dipteran populations (Changbunjong et al., 2020; Chaiphongpachara et al., 2022a; Yildirim et al., 2024). Together, these findings provide baseline information for future studies on population variation, ecology, and management of *H. exigua* in Thailand.

A limitation of this study is that each geographical region was represented by a single farm/locality, while host type, sampling period, and local conditions varied among sites. In addition, multiple flies were collected from the same host animals, which may have introduced some non-independence among specimens. Therefore, the effects of geography, farm characteristics, host type, environment, and host-level sampling could not be fully separated. The observed genetic and wing morphometric differences should therefore be regarded as variation among the sampled populations rather than as effects of geographical region alone. Future studies including multiple farms, greater host-level replication, and standardized sampling will help clarify the relative contribution of these factors.

## 5 Conclusions

This study provides the first combined assessment of genetic and wing phenotypic variation among *H. exigua* populations in Thailand. The findings indicate relatively high mitochondrial haplotype diversity and limited population genetic structure, characterized by generally low genetic differentiation among the sampled populations and a pattern consistent with possible demographic expansion. Wing GM analyses also revealed significant variation in wing shape among the sampled populations. The molecular and morphometric approaches provide complementary population-level information on genetic and phenotypic variation in *H. exigua* and establish baseline information for future studies on its ecology, dispersal, and management in Thailand.

## Ethical approval

All animal procedures were reviewed and approved by the Animal Care and Use Committee of the Faculty of Veterinary Science at Mahidol University, Thailand (Ref. MUVS-2024-04-30).

## CRediT authorship contribution statement

**Tanasak Changbunjong:** Conceptualization, Investigation, Methodology, Formal analysis, Data curation, Writing - original draft, Writing - review & editing, Visualization, Supervision, Project administration, Funding acquisition. **Thekhawet Weluwanarak:** Conceptualization, Investigation, Methodology, Visualization, Formal analysis. **Sedthapong Laojun:** Conceptualization, Investigation, Methodology, Formal analysis. **Tanawat Chaiphongpachara:** Conceptualization, Investigation, Methodology, Formal analysis, Supervision, Visualization, Writing - original draft, Writing - review & editing.

## Statement on the use of AI-assisted technologies

During the preparation of this manuscript, the authors used ChatGPT (OpenAI) to assist with checking grammar, sentence structure, and language clarity. All content was subsequently reviewed and edited by the authors, who take full responsibility for the final version of the publication.

## Funding

This research was funded by 10.13039/501100004156Mahidol University (Fundamental Fund: fiscal year 2026 by 10.13039/100017413National Science Research and Innovation Fund (NSRF)), grant number FF-160/2569.

## Declaration of competing interests

The authors declare that they have no known competing financial interests or personal relationships that could have appeared to influence the work reported in this paper.

## Data Availability

The data supporting the conclusions of this article are included within the article and its supplementary file. The newly generated sequences were submitted to the GenBank database under the accession numbers PZ631306-PZ631369.
