# Supplementary material for: Mitochondrial cytochrome b sequence variation and wing geometric morphometrics among Haematobia exigua (Diptera: Muscidae) populations in Thailand
Source: Curr Res Parasitol Vector Borne Dis. 2026 Oct 9;10:100446. doi: 10.1016/j.crpvbd.2026.100446 (PMC13697330; doi:10.1016/j.crpvbd.2026.100446)
Supplement: Supplementary file 1 [file mmc1.pdf]

Supplementary Table S1. BLAST comparison of 64 mitochondrial cytochrome *b* (*cytb*) sequences of *Haematobia exigua* generated in this study with reference sequences in GenBank.

GenBank accession of study sequence	Closest BLAST match	Reference accession	Identity (%)	Query coverage (%)
PZ631306	*Haematobia exigua*	KU600012	100	94
PZ631307	*Haematobia exigua*	KJ470650	99.64	94
PZ631308	*Haematobia exigua*	KJ470650	100	94
PZ631309	*Haematobia exigua*	KU599993	100	94
PZ631310	*Haematobia exigua*	KJ470650	99.82	94
PZ631311	*Haematobia exigua*	KJ470650	100	94
PZ631312	*Haematobia exigua*	KJ470650	99.82	94
PZ631313	*Haematobia exigua*	KJ470650	99.82	94
PZ631314	*Haematobia exigua*	KJ470650	100	94
PZ631315	*Haematobia exigua*	KJ470650	100	94
PZ631316	*Haematobia exigua*	KU599993	100	94
PZ631317	*Haematobia exigua*	KJ470650	100	94
PZ631318	*Haematobia exigua*	KJ470650	100	94
PZ631319	*Haematobia exigua*	KJ470650	99.82	94
PZ631320	*Haematobia exigua*	KJ470650	100	94
PZ631321	*Haematobia exigua*	KJ470650	100	94
PZ631322	*Haematobia exigua*	KJ470650	99.82	94
PZ631323	*Haematobia exigua*	KU599993	100	94
PZ631324	*Haematobia exigua*	KJ470650	100	94
PZ631325	*Haematobia exigua*	KU599993	100	94
PZ631326	*Haematobia exigua*	KU600011	100	94
PZ631327	*Haematobia exigua*	KJ470650	100	94
PZ631328	*Haematobia exigua*	KJ470650	100	94
PZ631329	*Haematobia exigua*	KJ470650	100	94
PZ631330	*Haematobia exigua*	KU600012	100	94
PZ631331	*Haematobia exigua*	KJ470650	100	94
PZ631332	*Haematobia exigua*	KU599993	100	94
PZ631333	*Haematobia exigua*	KJ470650	100	94
PZ631334	*Haematobia exigua*	KU600012	100	94
PZ631335	*Haematobia exigua*	KU600011	100	94
PZ631336	*Haematobia exigua*	KU600012	100	94
PZ631337	*Haematobia exigua*	KJ470650	100	94
PZ631338	*Haematobia exigua*	KU599993	99.64	94
PZ631339	*Haematobia exigua*	KJ470650	100	94
PZ631340	*Haematobia exigua*	KJ470650	100	94
PZ631341	*Haematobia exigua*	KJ470650	100	94
PZ631342	*Haematobia exigua*	KJ470650	100	94

GenBank accession of study sequence	Closest BLAST match	Reference accession	Identity (%)	Query coverage (%)
PZ631343	*Haematobia exigua*	KJ470650	100	94
PZ631344	*Haematobia exigua*	KJ470650	100	94
PZ631345	*Haematobia exigua*	KJ470650	99.82	94
PZ631346	*Haematobia exigua*	KJ470650	100	94
PZ631347	*Haematobia exigua*	KJ470650	100	94
PZ631348	*Haematobia exigua*	KJ470650	100	94
PZ631349	*Haematobia exigua*	KU599993	100	94
PZ631350	*Haematobia exigua*	KJ470650	100	94
PZ631351	*Haematobia exigua*	KJ470650	100	94
PZ631352	*Haematobia exigua*	KJ470650	100	94
PZ631353	*Haematobia exigua*	KJ470650	99.82	94
PZ631354	*Haematobia exigua*	KJ470650	99.82	94
PZ631355	*Haematobia exigua*	KU599999	99.82	94
PZ631356	*Haematobia exigua*	KJ470650	99.82	94
PZ631357	*Haematobia exigua*	KU600011	99.46	94
PZ631358	*Haematobia exigua*	KJ470650	100	94
PZ631359	*Haematobia exigua*	KU599988	100	94
PZ631360	*Haematobia exigua*	KJ470650	100	94
PZ631361	*Haematobia exigua*	KJ470650	99.82	94
PZ631362	*Haematobia exigua*	KJ470650	99.64	94
PZ631363	*Haematobia exigua*	KJ470650	100	94
PZ631364	*Haematobia exigua*	KJ470650	100	94
PZ631365	*Haematobia exigua*	KJ470650	100	94
PZ631366	*Haematobia exigua*	KJ470650	100	94
PZ631367	*Haematobia exigua*	KU599988	100	94
PZ631368	*Haematobia exigua*	KJ470650	100	94
PZ631369	*Haematobia exigua*	KJ470650	100	94
